# 2,10-Dibromo-6,6-dimethyl­dibenzo[*d*,*f*][1,3]dioxepine

**DOI:** 10.1107/S1600536808007186

**Published:** 2008-03-20

**Authors:** Hai-Quan Zhang, Bao Li, Guang-Di Yang, Yu-Guang Ma

**Affiliations:** a State Key Laboratory of Metastable Materials Science and Technology, Yanshan University, Qinhuangdao 066004, People’s Republic of China; b State Key Laboratory of Supramolecular Structure and Materials, Jilin University, Changchun 130012, People’s Republic of China

## Abstract

In the crystal structure of the title compound, C_15_H_12_Br_2_O_2_, which was synthesized from 2,10-dibromo-2,2′-dihydroxy­biphenyl and 2,2-dimethoxy­propane, the aromatic rings are twisted by 35 (1)°. The heterocyclic ring exhibits a twisted conformation.

## Related literature

For background literarture on dibenzo[*d*,*f*][1,3]dioxepine derivatives, see: Dean (1963 ▶). For applications, see: He *et al.* (2003 ▶). For the synthesis of the title compound, see: Zhang *et al.* (2003 ▶).
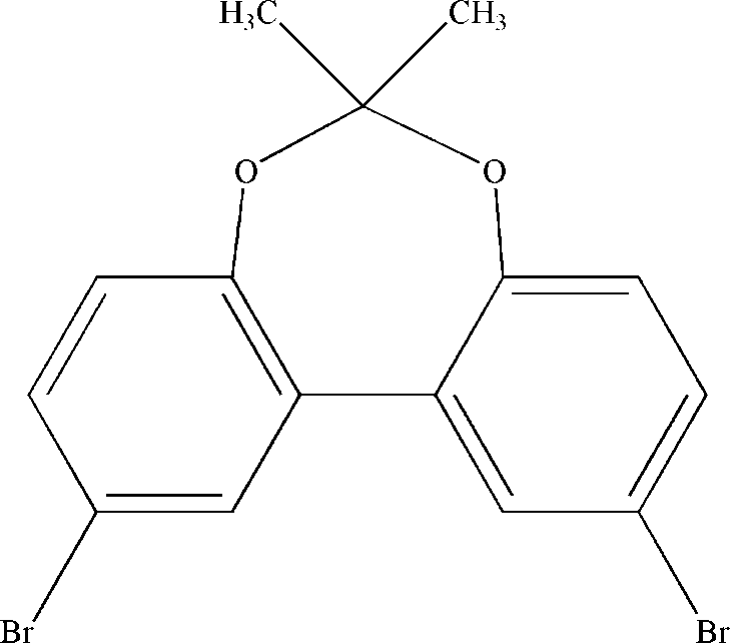


## Experimental

### 

#### Crystal data

C_15_H_12_Br_2_O_2_
*M* *_r_* = 384.07
Monoclinic, 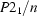

*a* = 10.8411 (6) Å
*b* = 7.6902 (3) Å
*c* = 16.7466 (8) Å
β = 99.824 (2)°
*V* = 1375.70 (11) Å^3^
*Z* = 4
Mo *K*α radiation
μ = 5.89 mm^−1^
*T* = 291 (2) K
0.07 × 0.06 × 0.06 mm

#### Data collection

Rigaku R-AXIS RAPID diffractometer
Absorption correction: multi-scan (*ABSCOR*; Higashi, 1995 ▶) *T*_min_ = 0.690, *T*_max_ = 0.726
5409 measured reflections
3145 independent reflections
2211 reflections with *I* > 2σ(*I*)
*R*_int_ = 0.023

#### Refinement

*R*[*F* ^2^ > 2σ(*F* ^2^)] = 0.023
*wR*(*F* ^2^) = 0.058
*S* = 0.94
3145 reflections
174 parameters
H-atom parameters constrained
Δρ_max_ = 0.27 e Å^−3^
Δρ_min_ = −0.29 e Å^−3^

### 

Data collection: *RAPID-AUTO* (Rigaku, 1998 ▶); cell refinement: *RAPID-AUTO*; data reduction: *CrystalStructure* (Rigaku/MSC, 2002 ▶); program(s) used to solve structure: *SHELXS97* (Sheldrick, 2008 ▶); program(s) used to refine structure: *SHELXL97* (Sheldrick, 2008 ▶); molecular graphics: *PLATON* (Spek, 2003 ▶); software used to prepare material for publication: *SHELXL97*.

## Supplementary Material

Crystal structure: contains datablocks global, I. DOI: 10.1107/S1600536808007186/ng2433sup1.cif

Structure factors: contains datablocks I. DOI: 10.1107/S1600536808007186/ng2433Isup2.hkl

Additional supplementary materials:  crystallographic information; 3D view; checkCIF report

## Figures and Tables

**Fig. 1.**
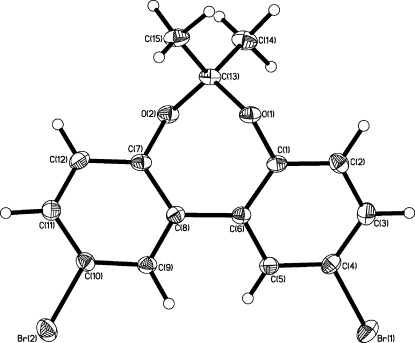
The structure of the title compound, with the atom-labelling scheme. Displacement ellipsoids are drawn at the 30% probability level of arbitrary radii.

## supplementary crystallographic information

### Comment

Dibenzo[d,f][1,3]dioxepine derivatives is very important in pharmaceutical applications (Dean, 1963). In fact it has been found that such a structure, which is probably related to their pharmacological activiry, is present in many biologically active natural products. Introducing functional group Br on benzene ring of dibenzo[d,f][1,3] dioxepine can expand the field of their application, such as photoluminescence, electro-luminescence devices and nonlinear optics (. We have reported the synthesis of the 2,10-dibro-dimethyl- dibenzo[d,f][1,3]dioxepine (Zhang *et al.*, 2003).Herein we present the crysal structure of the title compound.

### Experimental

The 2,10-dibro-dimethyl-dibenzo[d,f][1,3]dioxepine was dissolved in ethanol.The solution was allowed to stand at room temperature for several day, white block-shaped crystal was obtained with slow volative solvent.

### Refinement

C-bound H atoms were geometrically positioned with C—H = 0.97 Å, *U*_iso_(H) = 1.5*U*_eq_(C) for methyl and C—H = 0.93 Å, *U*_iso_(H) = 1.2*U*_eq_(C) for other carbon atoms.

### Crystal data

**Table:**

C_15_H_12_Br_2_O_2_	*F*_000_ = 752
*M**_r_* = 384.07	*D*_x_ = 1.854 Mg m^−^^3^
Monoclinic, *P*2_1_/*n*	Mo *K*α radiation λ = 0.71073 Å
Hall symbol: -P 2yn	Cell parameters from 8634 reflections
*a* = 10.8411 (6) Å	θ = 2.5–54.9º
*b* = 7.6902 (3) Å	µ = 5.89 mm^−^^1^
*c* = 16.7466 (8) Å	*T* = 291 (2) K
β = 99.824 (2)º	Block, colorless
*V* = 1375.70 (11) Å^3^	0.07 × 0.06 × 0.06 mm
*Z* = 4	

### Data collection

**Table:**

Rigaku R-AXIS RAPID diffractometer	3145 independent reflections
Radiation source: fine-focus sealed tube	2211 reflections with *I* > 2σ(*I*)
Monochromator: graphite	*R*_int_ = 0.023
*T* = 291(2) K	θ_max_ = 27.5º
ω scans	θ_min_ = 2.1º
Absorption correction: multi-scan(ABSCOR; Higashi, 1995)	*h* = −14→14
*T*_min_ = 0.690, *T*_max_ = 0.726	*k* = −9→9
5409 measured reflections	*l* = −21→21

### Refinement

**Table:**

Refinement on *F*^2^	Secondary atom site location: difference Fourier map
Least-squares matrix: full	Hydrogen site location: inferred from neighbouring sites
*R*[*F*^2^ > 2σ(*F*^2^)] = 0.023	H-atom parameters constrained
*wR*(*F*^2^) = 0.058	*w* = 1/[σ^2^(*F*_o_^2^) + (0.0241*P*)^2^] where *P* = (*F*_o_^2^ + 2*F*_c_^2^)/3
*S* = 0.94	(Δ/σ)_max_ = 0.002
3145 reflections	Δρ_max_ = 0.27 e Å^−^^3^
174 parameters	Δρ_min_ = −0.29 e Å^−^^3^
Primary atom site location: structure-invariant direct methods	Extinction correction: none

### Special details

**Table:**

Geometry. All e.s.d.'s (except the e.s.d. in the dihedral angle between two l.s. planes) are estimated using the full covariance matrix. The cell e.s.d.'s are taken into account individually in the estimation of e.s.d.'s in distances, angles and torsion angles; correlations between e.s.d.'s in cell parameters are only used when they are defined by crystal symmetry. An approximate (isotropic) treatment of cell e.s.d.'s is used for estimating e.s.d.'s involving l.s. planes.
Refinement. Refinement of *F*^2^ against ALL reflections. The weighted *R*-factor *wR* and goodness of fit *S* are based on *F*^2^, conventional *R*-factors *R* are based on *F*, with *F* set to zero for negative *F*^2^. The threshold expression of *F*^2^ > σ(*F*^2^) is used only for calculating *R*-factors(gt) *etc*. and is not relevant to the choice of reflections for refinement. *R*-factors based on *F*^2^ are statistically about twice as large as those based on *F*, and *R*- factors based on ALL data will be even larger.

### Fractional atomic coordinates and isotropic or equivalent isotropic displacement parameters (Å^2^)

**Table:**

	*x*	*y*	*z*	*U*_iso_*/*U*_eq_	
Br1	0.86808 (3)	1.13273 (4)	0.551106 (16)	0.03688 (10)	
Br2	1.02801 (3)	0.90894 (4)	0.113685 (17)	0.03706 (10)	
C1	0.5935 (2)	0.8979 (3)	0.33413 (15)	0.0231 (6)	
C2	0.5678 (3)	0.8810 (4)	0.41168 (15)	0.0275 (6)	
H2	0.4948	0.8261	0.4202	0.033*	
C3	0.6512 (3)	0.9460 (4)	0.47671 (16)	0.0282 (7)	
H3	0.6350	0.9342	0.5292	0.034*	
C4	0.7584 (3)	1.0285 (4)	0.46290 (15)	0.0255 (6)	
C5	0.7875 (3)	1.0420 (4)	0.38539 (14)	0.0256 (6)	
H5	0.8616	1.0949	0.3773	0.031*	
C6	0.7041 (3)	0.9752 (4)	0.31986 (14)	0.0224 (6)	
C7	0.6336 (3)	1.0283 (4)	0.17277 (15)	0.0237 (6)	
C8	0.7306 (3)	0.9841 (4)	0.23570 (15)	0.0216 (6)	
C9	0.8488 (3)	0.9485 (3)	0.21775 (15)	0.0242 (6)	
H9	0.9145	0.9188	0.2588	0.029*	
C10	0.8673 (3)	0.9578 (4)	0.13799 (15)	0.0247 (6)	
C11	0.7722 (3)	1.0031 (4)	0.07585 (15)	0.0282 (7)	
H11	0.7869	1.0095	0.0228	0.034*	
C12	0.6549 (3)	1.0388 (4)	0.09347 (15)	0.0286 (7)	
H12	0.5901	1.0699	0.0521	0.034*	
C13	0.4400 (3)	0.9391 (4)	0.21344 (16)	0.0263 (7)	
C14	0.3410 (3)	1.0282 (4)	0.25113 (17)	0.0345 (7)	
H14A	0.2855	0.9428	0.2672	0.052*	
H14B	0.2944	1.1061	0.2125	0.052*	
H14C	0.3797	1.0927	0.2978	0.052*	
C15	0.3877 (3)	0.8282 (4)	0.14128 (16)	0.0339 (7)	
H15A	0.4553	0.7807	0.1179	0.051*	
H15B	0.3354	0.8981	0.1017	0.051*	
H15C	0.3392	0.7352	0.1583	0.051*	
O1	0.51386 (17)	0.8227 (2)	0.26952 (10)	0.0258 (4)	
O2	0.51830 (17)	1.0765 (2)	0.19107 (10)	0.0261 (4)	

### Atomic displacement parameters (Å^2^)

**Table:**

	*U*^11^	*U*^22^	*U*^33^	*U*^12^	*U*^13^	*U*^23^
Br1	0.0428 (2)	0.0392 (2)	0.02494 (13)	−0.00242 (16)	−0.00487 (12)	−0.00039 (14)
Br2	0.03242 (18)	0.0427 (2)	0.03910 (17)	0.00823 (16)	0.01491 (13)	0.00811 (15)
C1	0.0206 (14)	0.0192 (16)	0.0281 (13)	0.0013 (13)	0.0004 (11)	−0.0015 (12)
C2	0.0244 (15)	0.0257 (17)	0.0341 (14)	0.0030 (13)	0.0099 (12)	0.0030 (13)
C3	0.0309 (17)	0.0292 (18)	0.0255 (13)	0.0066 (14)	0.0072 (12)	0.0028 (12)
C4	0.0297 (17)	0.0210 (16)	0.0236 (13)	0.0043 (13)	−0.0017 (12)	−0.0003 (12)
C5	0.0206 (15)	0.0269 (17)	0.0289 (14)	−0.0012 (13)	0.0027 (12)	0.0015 (12)
C6	0.0222 (16)	0.0193 (16)	0.0247 (13)	0.0027 (12)	0.0014 (11)	0.0019 (11)
C7	0.0223 (16)	0.0178 (16)	0.0296 (14)	−0.0020 (12)	0.0004 (12)	−0.0009 (12)
C8	0.0218 (15)	0.0167 (15)	0.0254 (13)	−0.0026 (12)	0.0016 (11)	0.0016 (11)
C9	0.0212 (16)	0.0236 (17)	0.0261 (13)	−0.0033 (12)	−0.0002 (11)	0.0032 (11)
C10	0.0225 (16)	0.0225 (17)	0.0296 (14)	−0.0011 (13)	0.0058 (12)	−0.0002 (12)
C11	0.0302 (18)	0.0300 (18)	0.0242 (13)	−0.0056 (14)	0.0043 (13)	−0.0008 (13)
C12	0.0265 (17)	0.0311 (18)	0.0251 (14)	−0.0027 (14)	−0.0045 (12)	0.0028 (12)
C13	0.0190 (16)	0.0256 (18)	0.0325 (14)	−0.0018 (13)	−0.0009 (12)	−0.0018 (12)
C14	0.0241 (17)	0.035 (2)	0.0433 (17)	0.0016 (14)	0.0042 (14)	−0.0088 (15)
C15	0.0248 (17)	0.037 (2)	0.0376 (15)	−0.0035 (14)	−0.0011 (13)	−0.0103 (15)
O1	0.0213 (11)	0.0220 (11)	0.0323 (9)	−0.0027 (9)	−0.0002 (8)	−0.0019 (9)
O2	0.0209 (11)	0.0225 (12)	0.0337 (10)	0.0032 (9)	0.0007 (8)	0.0023 (9)

### Geometric parameters (Å, °)

**Table:**

Br1—C4	1.908 (3)	C9—C10	1.386 (3)
Br2—C10	1.893 (3)	C9—H9	0.9300
C1—C2	1.380 (3)	C10—C11	1.379 (4)
C1—O1	1.390 (3)	C11—C12	1.381 (4)
C1—C6	1.395 (4)	C11—H11	0.9300
C2—C3	1.385 (4)	C12—H12	0.9300
C2—H2	0.9300	C13—O1	1.438 (3)
C3—C4	1.378 (4)	C13—O2	1.444 (3)
C3—H3	0.9300	C13—C14	1.501 (4)
C4—C5	1.391 (3)	C13—C15	1.508 (4)
C5—C6	1.396 (3)	C14—H14A	0.9600
C5—H5	0.9300	C14—H14B	0.9600
C6—C8	1.487 (3)	C14—H14C	0.9600
C7—O2	1.387 (3)	C15—H15A	0.9600
C7—C12	1.389 (3)	C15—H15B	0.9600
C7—C8	1.398 (3)	C15—H15C	0.9600
C8—C9	1.393 (4)		
			
C2—C1—O1	119.7 (2)	C11—C10—Br2	119.0 (2)
C2—C1—C6	121.2 (3)	C9—C10—Br2	119.1 (2)
O1—C1—C6	118.8 (2)	C10—C11—C12	119.1 (2)
C1—C2—C3	119.8 (3)	C10—C11—H11	120.5
C1—C2—H2	120.1	C12—C11—H11	120.5
C3—C2—H2	120.1	C11—C12—C7	120.2 (3)
C4—C3—C2	119.4 (2)	C11—C12—H12	119.9
C4—C3—H3	120.3	C7—C12—H12	119.9
C2—C3—H3	120.3	O1—C13—O2	109.8 (2)
C3—C4—C5	121.6 (3)	O1—C13—C14	111.6 (2)
C3—C4—Br1	119.7 (2)	O2—C13—C14	105.6 (2)
C5—C4—Br1	118.7 (2)	O1—C13—C15	105.2 (2)
C4—C5—C6	119.1 (3)	O2—C13—C15	111.3 (2)
C4—C5—H5	120.5	C14—C13—C15	113.4 (2)
C6—C5—H5	120.5	C13—C14—H14A	109.5
C1—C6—C5	118.9 (2)	C13—C14—H14B	109.5
C1—C6—C8	119.5 (2)	H14A—C14—H14B	109.5
C5—C6—C8	121.6 (2)	C13—C14—H14C	109.5
O2—C7—C12	119.9 (2)	H14A—C14—H14C	109.5
O2—C7—C8	119.3 (2)	H14B—C14—H14C	109.5
C12—C7—C8	120.5 (3)	C13—C15—H15A	109.5
C9—C8—C7	119.1 (2)	C13—C15—H15B	109.5
C9—C8—C6	122.0 (2)	H15A—C15—H15B	109.5
C7—C8—C6	118.9 (2)	C13—C15—H15C	109.5
C10—C9—C8	119.2 (3)	H15A—C15—H15C	109.5
C10—C9—H9	120.4	H15B—C15—H15C	109.5
C8—C9—H9	120.4	C1—O1—C13	116.9 (2)
C11—C10—C9	121.9 (3)	C7—O2—C13	116.9 (2)
